# Intensive-Phase Treatment Outcomes among Hospitalized Multidrug-Resistant Tuberculosis Patients: Results from a Nationwide Cohort in Nigeria

**DOI:** 10.1371/journal.pone.0094393

**Published:** 2014-04-10

**Authors:** Olanrewaju Oladimeji, Petros Isaakidis, Olusegun J. Obasanya, Osman Eltayeb, Mohammed Khogali, Rafael Van den Bergh, Ajay M. V. Kumar, Sven Gudmund Hinderaker, Saddiq T. Abdurrahman, Lovett Lawson, Luis E. Cuevas

**Affiliations:** 1 Zankli Medical Centre, Abuja, Nigeria; 2 Liverpool School of Tropical Medicines, Pembroke Place, Liverpool, United Kingdom; 3 Médecins Sans Frontières (MSF), Operational Research Unit, Luxemburg, Luxemburg; 4 National Tuberculosis and Leprosy Control Unit, Federal Ministry of Health, Abuja, Nigeria; 5 Damien Foundation Belgium, Nigeria Project, Ibadan, Nigeria; 6 International Union Against Tuberculosis and Lung Disease (The Union), South-East Asia Regional Office, New Delhi, India; 7 Centre for International Health, University of Bergen, Bergen, Norway; 8 Tuberculosis and Leprosy Control Program, Federal Capital Territory, Abuja, Nigeria; McGill University, Canada

## Abstract

**Background:**

Nigeria is faced with a high burden of Human Immunodeficiency Virus (HIV) infection and multidrug-resistant tuberculosis (MDR-TB). Treatment outcomes among MDR-TB patients registered across the globe have been poor, partly due to high loss-to-follow-up. To address this challenge, MDR-TB patients in Nigeria are hospitalized during the intensive-phase(IP) of treatment (first 6–8 months) and are provided with a package of care including standardized MDR-TB treatment regimen, antiretroviral therapy (ART) and cotrimoxazole prophylaxis (CPT) for HIV-infected patients, nutritional and psychosocial support. In this study, we report the end-IP treatment outcomes among them.

**Methods:**

In this retrospective cohort study, we reviewed the patient records of all bacteriologically-confirmed MDR-TB patients admitted for treatment between July 2010 and October 2012.

**Results:**

Of 162 patients, 105(65%) were male, median age was 34 years and 28(17%) were HIV-infected; all 28 received ART and CPT. Overall, 138(85%) were alive and culture negative at the end of IP, 24(15%) died and there was no loss-to-follow-up. Mortality was related to low CD4-counts at baseline among HIV-positive patients. The median increase in body mass index among those documented to be underweight was 2.6 kg/m^2^ (p<0.01) and CD4-counts improved by a median of 52 cells/microL among the HIV-infected patients (p<0.01).

**Conclusions:**

End-IP treatment outcomes were exceptional compared to previously published data from international cohorts, thus confirming the usefulness of a hospitalized model of care. However, less than five percent of all estimated 3600 MDR-TB patients in Nigeria were initiated on treatment during the study period. Given the expected scale-up of MDR-TB care, the hospitalized model is challenging to sustain and the national TB programme is contemplating to move to ambulatory care. Hence, we recommend using both ambulatory and hospitalized approaches, with the latter being reserved for selected high-risk groups.

## Introduction

Multidrug-resistant tuberculosis (MDR-TB) is a growing public health problem that jeopardizes the progress made in tuberculosis care and control worldwide [1], [2]. Globally, the prevalence of MDR-TB is estimated to be 3.1% among new TB patients and 10% among previously treated patients [2]. In Nigeria, the prevalence of MDR-TB is reported to be 2.9% among newly detected cases and 14.5% in previously treated cases and the number of MDR-TB patients is estimated to be between 2700 and 4500 [3], [4].

In Nigeria, the prevalence of HIV is estimated to be 4.4% in the general population and that among TB patients has increased from 2.2% in 1991 to about 27% in 2008[5]. The prevalence of HIV/MDR-TB co-infection in Nigeria, however, is not known to-date. An individual patient data meta-analysis by Ahuja et al, showed that “global MDR-TB treatment outcomes were poor: treatment success was achieved in only slightly more than half of the patients” [6]. Treatment success was associated with overall duration of treatment, number of effective drugs in the regimen, and with use of later generation fluoroquinolones (levofloxacin, moxifloxacin, gatifloxacin, and sparfloxacin)” but no association with hospitalization or ambulatory approach during the intensive-phase was examined. Several studies from settings in different parts of the world reported 77 to 88% of patients converted sputum culture to negative after a median of 2 to 3 months of treatment initiation [6]–[9]. However, with the exception of South Africa, very limited data are available on intensive-phase and completed treatment outcomes from African cohorts.

There is ample evidence that ambulatory strategies for the treatment of MDR-TB are considerably effective and use fewer resources which is a major issue in countries with high disease burden and limited resources [10]. The World Health Organization (WHO) recommends ambulatory treatment for MDR-TB and most national TB programmes have adopted an ambulatory strategy. However, the National Tuberculosis Program in Nigeria opted for an in-patient approach to allow for better adherence, adequate monitoring of adverse reactions, regular sputum smear and culture examination, provision of antiretroviral treatment (ART) to HIV co-infected patients and multi-disciplinary attention to the patients.

Our study aimed to assess the intensive-phase treatment outcomes among all MDR-TB in-patients receiving treatment in Nigeria. The specific objectives of the study were: 1) to describe the clinical and demographic characteristics of the patients of the Nigerian MDR-TB cohort; 2) to determine survival and smear and culture conversion by the end of the intensive-phase of treatment, stratified by HIV-status; and 3) to explore associations between selected risk factors and treatment outcomes in this cohort.

## Methods

### Ethics

Ethical approval was given by National Health Research Ethics Committee of Nigeria in June, 2013. This study has also met the Médecins Sans Frontières Ethics Review Board (Geneva, Switzerland) approved criteria for analysis of routinely-collected program data in May, 2013. It satisfies the requirements of the Ethics Advisory Group of the International Union Against Tuberculosis and Lung Disease, Paris, France. Patient information was anonymized and de-identified prior to analysis. As this was a routinely collected program data, informed consent from the patients was not obtained. The named ethics committees approved the study and waived the need for consent.

### Study design

This was a retrospective cohort study using routinely collected program data.

### Setting and study population

Nigeria, the largest country in the western region of Africa is a federation of 36 states and the Federal Capital Territory (FCT) with 774 administrative units referred to as-Local Government-Areas. The country has an estimated population of 154,729,000 by WHO in 2009 [5]. More than half of Nigerians (54.4%) live in poverty in spite of the huge revenues accruing from oil and gas. The country is composed of more than 250 ethnic groups with Yoruba, Igbo and Hausa being the most influential.-The health system in Nigeria is structured along three levels, namely primary, secondary and tertiary corresponding to the level of government that are responsible for health-care services, local government, the state, and the federal respectively. The-public and private-sectors are partners in the delivery of health care throughout the country [5].

Nigeria established its National TB and Leprosy Control Program (NTBLCP) in 1989. The NTBLCP operates along the three levels of government: National, State and Local Government Areas, with coordinating offices at each level. Health facilities at the peripheral level are the operational units of DOTS services. As of 2009, there were 3,455 health facilities providing free TB and DOTS services in Nigeria [11].

Currently, there are seven hospitals for MDR treatment in Nigeria. Of them, only five were functional during the study period which included the following: University College Hospital, Ibadan with 23 beds, General Chest Hospital, Jericho-Ibadan, with 24 beds, Infectious Diseases Hospital, Yaba-Lagos, with 40 beds, Lawrence Henshaw Hospital, Calabar with 14 beds and National Tuberculosis and Leprosy Training School, Zaria with 40 beds. There were no other hospitals in the public or private health sector providing MDR-TB treatment at the time of the study.

Infection control measures at the hospital wards, including environmental and administrative measures, were designed to prevent nosocomial transmission. In general, there were ventilators in all the wards and staff used N95 respirator whenever they came in contact with the patients irrespective of the culture status. In one of the facilities, there were cubicles where patients with culture conversion were moved to avoid re-infection.

### Patient's admission process

All diagnosed MDR-TB patients were first registered on a waiting list, given the limitation in the availability of hospital beds. Patients were notified by phone or during home visits as and when beds became available. Those patients who fully understood the need for long hospitalization and signed an informed consent form were finally admitted and initiated on treatment. The cost for hospital stay and treatment was covered by the programme.

### National MDR-TB treatment protocol

All bacteriologically confirmed MDR-TB patients received intensive phase for 6–8 months in the hospital, followed by 12 months of continuation phase in the community, based on the WHO updated guidelines in 2011[12]. A standardized treatment regimen was used, including five drugs: Kanamycin/Amikacin, Levofloxacin, Prothionamide, Cycloserine, Pyrazinamide (with Pyridoxine). Baseline investigations were done for patients admitted. Sputum smear microscopy and culture examinations were performed every month to monitor bacteriological response to treatment.

While the patients were admitted for MDR-TB treatment, they were supported by non-governmental organizations which provided complete nutritional support (three nutritionally balanced meals were served to each patient daily), stipends to make phone calls to their homes and provision of recreation activities such as games, books and films. Family and friends were allowed to visit in-patients over the weekend.

### Data collection and analysis

Demographic and clinical information of all MDR-TB patients admitted between July 2010 and October 2012 were extracted from the standardized program registers and double-entered into a Microsoft excel database, validated, subsequently imported into EpiData (version 2.2.2.182, EpiData Association, Odense, Denmark) for analysis.

The demographic and clinical characteristics of MDR-TB patients were described using proportions and median, as applicable. Chi-square test was used to compare differences in outcomes (death, culture conversion) between HIV/MDR-TB co-infected and non-co-infected patients. Survival curves were plotted for the time to sputum smear and culture conversion and time to death among the patients. We examined associations of sex, age, body mass index (BMI),CD4-count and HIV-status with adverse outcomes (defined as death and non-culture conversion) using chi square or Kruskal Wallis test as appropriate.

## Results

### Patient characteristics

Between July 2010 and October 2012, there were 162 bacteriologically confirmed MDR-TB patients admitted for treatment in five centres in Nigeria. Baseline and clinical characteristics of the patients in relation to their HIV-status are shown in **Table 1**. Sixty five per cent of the MDR-TB patients were males and median age was 34 years (Interquartile range (IQR): 29 – 42). About half (52%) of the patients were referred from the South West Zone of Nigeria.

**Table 1:** Demographic and clinical characteristics of hospitalized MDR-TB patients in Nigeria, July 2010-October 2012.

Variable	HIV-positive N (%)	HIV-negative N (%)	P-value
*Total*	28	134	
*Age(yr)*			
≤ 24	2 (7)	17 (13)	0.3
25 – 44	23 (82)	89 (66)	
≥ 45	3 (11)	28 (21)	
*Sex*			
Male	22 (79)	83 (62)	0.09
Female	6 (21)	51 (38)	
*Registration group*			
Failure of 1^st^ Treatment	5 (18)	13 (10)	0.2
Failure of Re-treatment	23 (82)	121 (90)	
*Pre-treatment BMI (kg/m^2^)*			
Underweight (< 18.5)	13 (46)	61 (46)	0.7
Normal/overweight (≥ 18.5)	13 (46)	70 (52)	
Unknown	2 (7)	3 (2)	
Pre-treatment CD4 count (cells/microL) *			
< 350	8 (29)	NA	NA
350 – 499	14 (50)	NA	
≥ 500	6 (21)	NA	

Twenty-eight (17%)of all the MDR-TB patients were co-infected with HIV, and over the course of the second line TB treatment all were on cotrimozaxole prophylaxis and antiretroviral treatment. Approximately half (46%) were underweight on admission. No statistical differences were observed in demographic and baseline clinical characteristics between HIV-infected and uninfected patients.

### End of intensive-phase treatment outcomes

#### Survival

**Figure 1:**
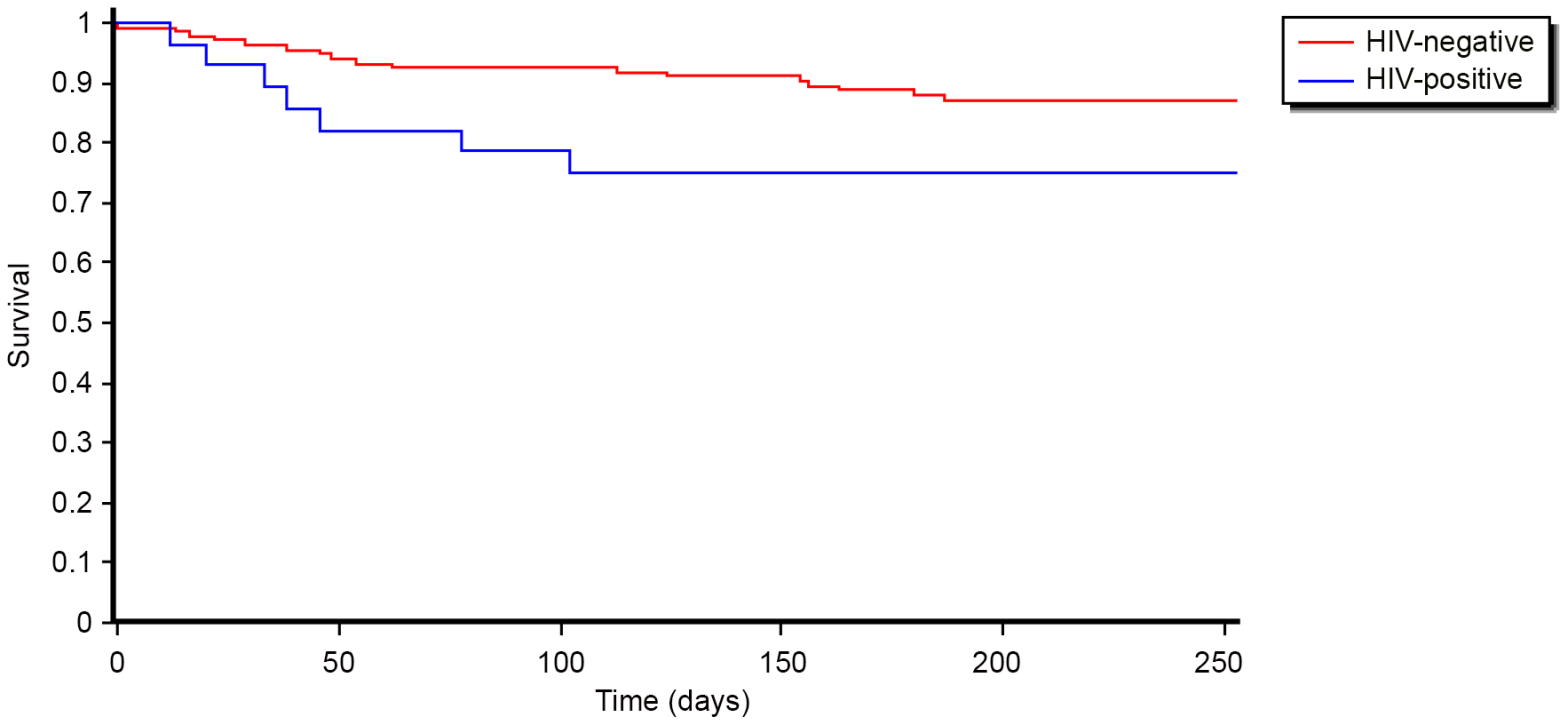
Survival among hospitalized MDR-TB patients stratified by HIV-status in Nigeria, July 2010 – October 2012.

Among all patients initiated on treatment, 24(15%) patients died and the remaining 138 (85%) were alive and on treatment at the end of the intensive phase. No patients were lost-to-follow-up in the Nigerian MDR-TB cohort. The timing of the deaths is indicated in **Figure 1**: the survival curve reflects early mortality in the first few weeks of treatment, and an overall higher proportion of deaths occurred among the HIV co-infected patients. After testing for associations with baseline socio-demographic and clinical factors, only baseline CD4-count was found to be significantly associated with death (median difference; 109 cells/microL, P<0.03).

#### Smear and culture conversion

**Figure 2:**
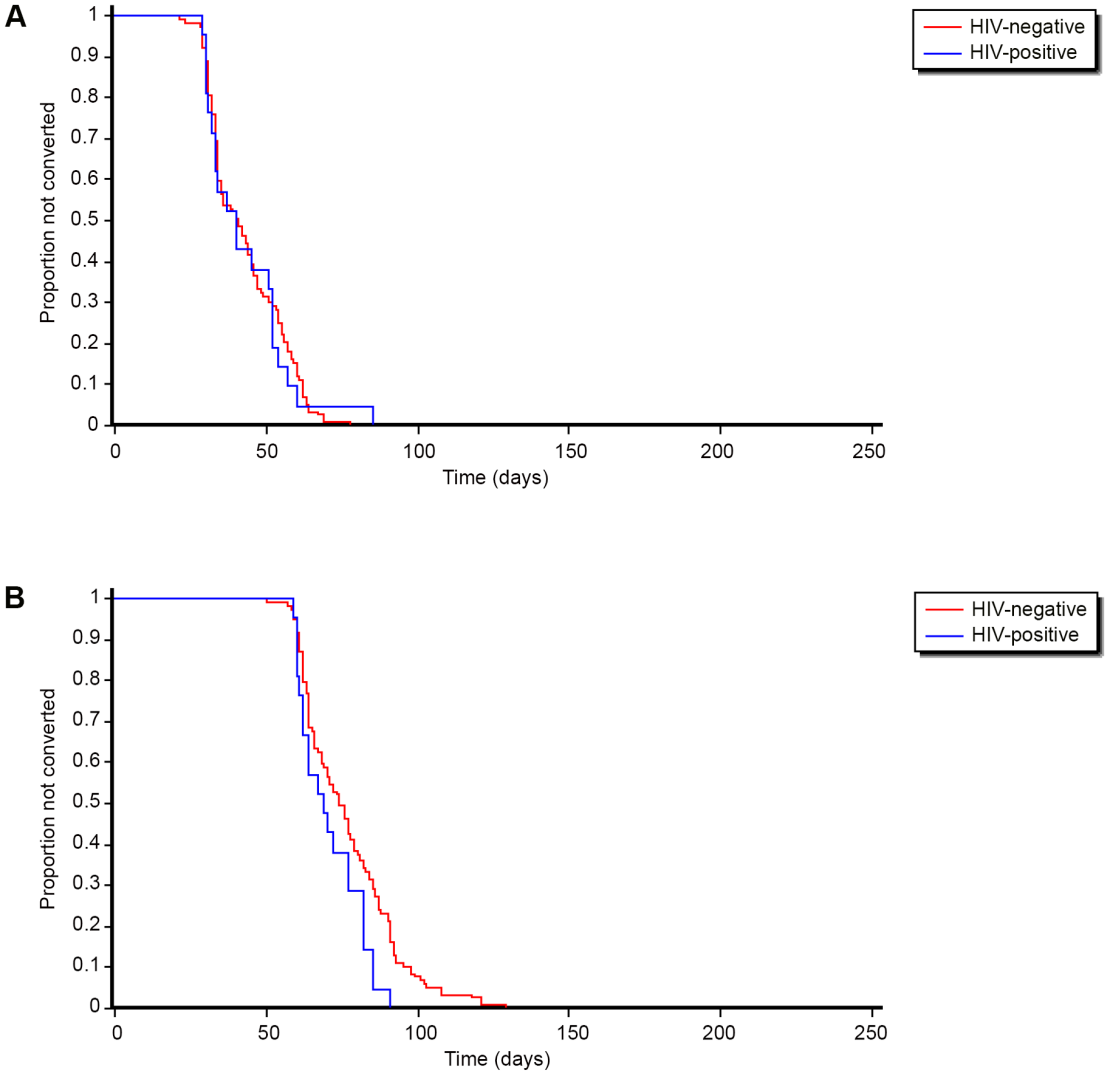
**a**. Time to sputum smear conversion among hospitalized MDR-TB patients stratified by HIV-status in Nigeria, July 2010 – October 2012. **b**.Time to culture conversion among hospitalized MDR-TB patients stratified by HIV-status in Nigeria, July 2010 – October 2012.

All of the 138 patients who were alive at the end of intensive phase had become sputum smear and culture negative. Kaplan-Meier curves showing the timing of sputum smear and culture conversion, stratified by HIV-status, are shown in **Figure 2**.There was no statistically significant difference in the time to sputum smear and culture conversion among HIV positive and negative individuals.

#### Clinical/nutritional and immunological recovery

Seventy four (46%) of the patients were underweight at the time of treatment initiation compared to 15 (9%) at the end of hospitalisation. Among patients who were underweight at baseline, a median gain in BMI of 2.6 Kg/m^2^ and 3.3 Kg/m^2^ was noted among HIV-positive and HIV-negative patients, respectively. Among HIV patients the median CD4-counts were 415 cells/microL at baseline and 491 cells/microL at six months. A median (IQR) CD4-count increase of 52 cells/mircoL (44–71) was observed, shown in **Table 2**.

**Table 2:** End of Intensive phase treatment outcomes of hospitalized MDR–TB patients in Nigeria July 2010 – October, 2012.

Outcome	HIV-positive N (%)	HIV-negative N (%)	P-value
Clinical Outcomes			
Alive, on treatment and culture negative	21 (75)	117 (87)	0.1
Dead	7 (25)	17 (13)	
Loss to follow-up	0	0	
BMI gained among underweight patients at baseline (Kg/m^2^)			
Median (IQR)	2.6 (1.8–2.9)	3.3 (2.5–4.4)	0.01
CD4- count gained(cells/microL)			
Median (IQR)	52 (42–71)	N/A	N/A

## Discussion

Based on a review of the current literature, we noted that only a limited number of MDR-TB cohorts from Africa have been described, all of which from South Africa [6], [13], . This is the first report on MDR-TB treatment outcomes from Nigeria, a country with a large burden of TB and a large number of MDR-TB patients (based on the WHO estimates). This national cohort is relatively small, but we report successful results in terms of survival, culture conversion and retention in care among HIV-infected and uninfected patients who were hospitalized throughout the intensive phase of treatment. We recorded no losses to follow-up in this cohort, a significant finding. Moreover, satisfactory clinical and immunological outcomes were recorded among HIV co-infected patients.

There was a trend towards overall poorer outcomes in HIV-infected individuals, with significant mortality in the first few weeks after treatment initiation; these results were consistent with previous studies showing increased mortality in HIV-positive compared to HIV-negative patients with drug-susceptible TB [13]–[15]. However, this difference was not found to be statistically significant owing to small numbers.

### Programme achievements

The end of intensive-phase treatment outcomes recorded so far in the program demonstrates the effectiveness of treating MDR-TB patients in a hospital setting. Some unique findings of this study included the exceptionally high retention in care among all patients and high up-take of ART among HIV co-infected patients (both 100%). While the exact reasons for this achievement are not clear, we speculate that the following factors may have contributed to these results: first, all patients received fully supervised DOT throughout the course of the intensive phase as this was feasible in a hospital setting. Second, there was intensive monitoring, early diagnosis of adverse events (including systematic and on-request clinical and laboratory assessment) and prompt management. Third, a full package of psychosocial services was offered to all hospitalized patients, which included nutritional support, individual counselling, and opportunities for recreational activities. We further suggest that the hospitalization (isolation) of patients and especially during the early days of treatment may have contributed to prevention of spread of the resistant strains to the community.

### Ambulatory and Hospital-based treatment strategies and policy implications

The study findings revoke the debate between hospitalized and ambulatory models of care for MDR-TB patients. According to the WHO, “the choice between hospitalization and ambulatory treatment depends on several factors in addition to the severity of the disease. Such factors include the availability of hospital beds with adequate infection control measures, the availability of trained personnel to administer treatment and manage adverse drug reactions; a social support network to facilitate adherence to ambulatory treatment; and the presence of other clinical or social conditions for in-patients.” [16]

In most settings, the community-based model of treatment is more feasible owing to the resource constraints faced by high TB burden countries [10], [17]–[19]. However, a major challenge in these models is the high loss-to-follow up reported [10], [17]–[19]. In our setting we have recorded no losses to follow-up, thus favouring the ‘hospitalization’ approach for treating MDR-TB patients. But this might be due to the relatively small scale of the programme and a highly selected cohort of patients. We hypothesize that the retention in care and treatment adherence may not be similar during the continuation (ambulatory) phase of treatment. Findings from countries of Eastern Europe with similar approaches to hospitalization during the intensive phase indicate a high treatment adherence during hospitalization, but this high adherence was not sustained in the ambulatory, continuation phase [20].

WHO estimates there are currently about 3600 MDR-TB cases annually among the notified pulmonary TB patients in Nigeria. Of this, only 162 (<5%) patients have been treated hitherto and this could be partially due to the limited capacity of the hospitalization model. This is further substantiated by the fact that a number of MDR-TB patients are in the waiting-list for treatment, due to lack of hospital beds.

Nigeria is rapidly expanding the provision of the new Xpert MTB/RIF test and, given that this test automatically tests for rifampicin resistance, it is expected that the need for hospital beds will soon increase exponentially. Mandatory hospitalization may become a major bottleneck for rapid scale-up of treatment unless resources are immediately allocated to increase capacity both in terms of infrastructure and in terms of trained human resources. Currently, the Nigerian National Tuberculosis Programme (NTP) is moving towards an ambulatory approach for both intensive and continuation phase in response to this anticipated increase. This approach will also require considerable resources in order to maintain such good outcomes. Despite all these limitations of a hospitalized model of care, the fact remains that this is effective in ensuring patient compliance to treatment, a major challenge in ambulatory care. Hence, it could be possible to adopt the best of both models of care with hospitalized approach used for select groups of patients with known high risk of loss to follow-up and mortality (like HIV-infected MDR-TB patients).

Based on the existing experiences from settings that have opted for ambulatory strategies and which are characterized by high loss to follow-up, we call for increased resources and innovative support strategies, including a patient-centred approach which includes nutritional and psychosocial support, in the management of MDR-TB patients.

### Limitations

There are several limitations to our study. First, the size of this observational cohort is relatively small. However, this represents all patients treated in Nigeria during the study period. Further, most MDR-TB cohorts reported till date are of small size. Second, we acknowledge that we only report on interim rather than final treatment outcomes, but still we think our data may contribute to the knowledge base. Third we acknowledge that this may have been a highly selective cohort with only those patients consenting to be hospitalized for the entire duration for intensive phase included in the study cohort; the selection procedure precluded the representativeness of our study populations. Fourth, as we report on a treatment cohort and not on-diagnosis cohort the adverse events may be significantly underestimated. It is documented that pre-treatment mortality and losses to follow-up are very high among HIV patients [21]–[27]. This was compounded by the strict selection procedure described.

Despite these limitations, this report provides some important evidence on the effectiveness, especially in terms of retention in care, of a hospital-based MDR-TB treatment model in HIV-infected and uninfected patients. Moreover, it provides insights that feed into the ongoing discussions about effectiveness and scalability.

### Conclusions, policy implications

The current approach to MDR-TB care in Nigeria which includes hospitalization in IP was found to be effective in ensuring treatment adherence with no loss to follow-up and high proportion alive and culture negative at the end of the IP. However, given that only less than 5% of all estimated MDR-TB patients are currently initiated on treatment and the expected increase in demand for hospital beds owing to implementation of Xpert MTB-RIF and rapid increases in MDR-TB case detection, this model may be challenging to sustain. Nigerian NTP, thus is contemplating to move to the model of ambulatory treatment. However, we recommend using both ambulatory and hospitalized approaches, with the latter being reserved for MDR-TB patients with higher risk of poor outcomes. Policy makers and clinicians should collaborate to find the best trade-off between excellent retention in care and the need for massive and fast scale-up of treatment, given high disease burden and resource constraints in Nigeria.
